# Reducible tungsten(VI) oxide-supported ruthenium(0) nanoparticles: highly active catalyst for hydrolytic dehydrogenation of ammonia borane

**DOI:** 10.55730/1300-0527.3607

**Published:** 2023-09-28

**Authors:** Serdar AKBAYRAK, Yalçın TONBUL, Saim ÖZKAR

**Affiliations:** 1 Department of Basic Sciences, Faculty of Engineering, Necmettin Erbakan University, Konya, Turkiye; 2 Ziya Gökalp Faculty of Education, Dicle University, Diyarbakır, Turkiye; 3 Department of Chemistry, Middle East Technical University, Ankara, Turkiye

**Keywords:** Ruthenium, tungsten(vi) oxide, catalytic activity, ammonia borane, reusability, hydrolytic dehydrogenation

## Abstract

Reducible WO_3_ powder with a mean diameter of 100 nm is used as support to stabilize ruthenium(0) nanoparticles. Ruthenium(0) nanoparticles are obtained by NaBH_4_ reduction of ruthenium(III) precursor on the surface of WO_3_ support at room temperature. Ruthenium(0) nanoparticles are uniformly dispersed on the surface of tungsten(VI) oxide. The obtained Ru^0^/WO_3_ nanoparticles are found to be active catalysts in hydrolytic dehydrogenation of ammonia borane. The turnover frequency (TOF) values of the Ru^0^/WO_3_ nanocatalysts with the metal loading of 1.0%, 2.0%, and 3.0% wt. Ru are 122, 106, and 83 min^−1^, respectively, in releasing hydrogen gas from the hydrolysis of ammonia borane at 25.0 °C. As the Ru^0^/WO_3_ (1.0% wt. Ru) nanocatalyst with an average particle size of 2.6 nm provides the highest activity among them, it is extensively investigated. Although the Ru^0^/WO_3_ (1.0% wt. Ru) nanocatalyst is not magnetically separable, it has extremely high reusability in the hydrolysis reaction as it preserves 100% of initial catalytic activity even after the 5th run of hydrolysis. The high activity and reusability of Ru^0^/WO_3_ (1.0% wt. Ru) nanocatalyst are attributed to the favorable metal-support interaction between the ruthenium(0) nanoparticles and the reducible tungsten(VI) oxide. The high catalytic activity and high stability of Ru^0^/WO_3_ nanoparticles increase the catalytic efficiency of precious ruthenium in hydrolytic dehydrogenation of ammonia borane.

## 1. Introduction

Due to depletion of energy reserves [1,2] and global warming mainly caused by the increasing atmospheric CO_2_ concentration [3], there exists an urgent request to substitute fossil fuels for the renewable energy sources on the way towards sustainable energy future [4,5]. As a green energy carrier, hydrogen is expected to have a vital role in this transition [6–8]. Conversely, the failure of safe and efficient storage of H_2_ gas impedes its large-scale application [9–11]. Fortunately, ammonia borane (AB, NH_3_BH_3_) appears to be one of the most promising solid hydrogen storage compounds thanks to its high hydrogen content, nontoxicity, lasting stability in liquid phase and solid state, and high water solubility [12–15]. Hydrolysis of AB has been found to be the most appropriate process for liberating H_2_ gas (Equation 1) [16–20].


(1) $${\text{H}}_{3}\text{N}\cdot \text{B}{\text{H}}_{3}(aq)+2{\text{H}}_{2}\text{O}(l)\overset{catalyst}{\to }\text{N}{\text{H}}_{4}^{+}(aq)+\text{B}{\text{O}}_{2}^{-}(aq)+3{\text{H}}_{2}(g)$$

Hydrolysis of AB is to be accelerated by transition metal nanocatalysts [21,22]. In particular, the nanoparticles (NPs) of platinum [23], palladium [24], and rhodium [25] are found to be the highest activity catalysts in releasing H_2_ from AB which can be used in pure hydrogen supply for on-board applications. Ruthenium is one of the most extensively tested noble metals for catalyzing the hydrolysis of AB [26]. Because of the relatively high price of ruthenium [27], the challenge is to increase its overall utilization efficiency in the hydrolytic dehydrogenation of AB. Although some water-soluble ruthenium complexes [28–30] are known to act as homogenous catalysts for releasing H_2_ from AB, the colloidal ruthenium(0) NPs provide quite high activity for the same reaction at room temperature [31–38]. However, the instability of transition metal NPs against agglomeration greatly hampers their catalytic performance [39]. Therefore, ruthenium(0) NPs have been stabilized by anchoring them on a variety of supports with large surface area [21,40]. Thus, ruthenium(0) NPs have been anchored on carbonaceous materials with large surface area [41–63], oxide nanopowders such as silica [64–66], cobalt oxide [67,68], molybdenum oxide [69], perovskite [70], alumina [71–73], titania [74–76], zirconia [77], hafnia [78], ceria [79], and xonotlite [80] and confined in the pores of zeolite [81–84] and metal-organic frameworks [85–90]. Using reducible oxide supports has been shown to provide noticeably high turnover frequency (TOF) for the Ru^0^ NPs in releasing H_2_ from AB: TOF = 241 min^−1^ on TiO_2_ [76], 173 min^−1^ on ZrO_2_ [77], 170 min^−1^ on HfO_2_ [78], 361 min^−1^ on CeO_2_ [79], and 2114 min^−1^ on Co_3_O_4_ [67]. WO_3_ is another unique reducible oxide which can increase the catalytic activity of transition metal NPs in hydrolytic dehydrogenation [91,92]. Particularly, using the transition metal(0) NPs on the oxygen-deficient WO_3−x_ can significantly increase the catalytic activity [93–96]. A 2019 paper [96] reports the use of rhodium(0) NPs on WO_3−x_ nanowires as catalysts for the hydrolysis of AB though under visible light irradiation. For the hydrogen evolution from AB, a significant enhancement in catalytic activity of nickel nanocatalyst has been achieved through its coupling with WO_3−x_ nanorods [97]. A very recent paper [98] reports the promotion effects of WO_3_ on the catalytic activity of platinum(0) NPs in the same reaction. We have also reported the use of WO_3_ as support for the rhodium(0) NPs in hydrogen evolution from AB [99]. The Rh^0^/WO_3_ nanocatalysts could be obtained by sodium borohydride reduction of rhodium(III) precursor impregnated on oxide powder. The resulting Rh^0^/WO_3_ (0.5% wt. Rh) nanocatalyst provides the highest TOF of 749 min^−1^ in liberating 3 equivalent H_2_ from the hydrolysis of AB at 25 °C [99]. The observed high catalytic activity is accredited to reducible nature of the oxide support. Herein, we report the use of WO_3_ support for the ruthenium(0) NPs and the employment of Ru^0^/WO_3_ in releasing H_2_ from AB. The Ru^0^/WO_3_ catalysts can be obtained by NaBH_4_ reduction of ruthenium(III) precursor impregnated on the oxide support and provide high activity in releasing H_2_ from AB.

## 2. Experimental

The sections on materials and instrumentation are given in Supplementary Information (SI).

### 2.1. Preparation of Ru/WO_3_

In a beaker, 1.0 g of WO_3_ was stirred with aqueous RuCl_3_.3H_2_O (54.6 mg) in distilled H_2_O (100 mL) for 24 h at room temperature. Next, ruthenium(III) ions were reduced with aqueous NaBH_4_ (mol NaBH_4_/mol Ru = 3). The resulting Ru^0^/WO_3_ NPs were isolated by centrifugation (10 min at 8000 rpm). The solid precipitate was washed with distilled H_2_O (15 mL) and separated again by centrifugation. The catalyst was then dried under vacuum at 60 °C for 12 h and characterized by various analytical techniques. The ruthenium content of the sample was determined as 2.0% wt. by ICP-OES analysis. Note that Ru^0^/WO_3_ (1.0% wt. Ru) and Ru^0^/WO_3_ (3.0% wt. Ru) samples were also prepared following the same procedure as the one given above by using 26.56 mg and 84.18 mg of RuCl_3_.3H_2_O, respectively. The Ru contents of the obtained samples were also determined by ICP-OES to be 1.0% and 3.01% wt. Ru, respectively.

### 2.2. Determination of the most active Ru loading for Ru/WO_3_ used in hydrolysis of AB

The catalytic activity of Ru^0^/WO_3_ (1.0% wt. Ru), Ru^0^/WO_3_ (2.0% wt. Ru), and Ru^0^/WO_3_ (3.0% wt. Ru) was tested in releasing H_2_ from the hydrolysis of AB starting with 0.60 mM Ru and 100 mM AB in 10 mL solution at 25.0 ± 0.1 °C. The highest activity was achieved by using Ru/WO_3_ (1.0% wt. Ru) which was used for all other tests.

### 2.3. Catalytic activity of Ru/WO_3_ in hydrolysis of AB at various conditions

The hydrolysis reaction was investigated as described in our previously published procedure [99], which was provided in the SI file. The reaction was performed starting with 60.0 mg of Ru/WO_3_ (1.0% wt. Ru) ([Ru] = 0.6 mM) in 10 mL H_2_O and AB (1.0 mmol) at different temperatures (20, 25, 30, 40 °C) in order to calculate the apparent activation energy. The catalytic activity of Ru/WO_3_ (1.0% wt) was also tested at different weights (30.0, 60.0, 90.0, 120.0 mg) and thus with various ruthenium concentrations (0.3, 0.6, 0.9, 1.18 mM) at 25 °C.

### 2.4. Durability of Ru/WO_3_ in hydrolysis of AB

Both recyclability and reusability of Ru/WO_3_ (1.0% wt.) catalyst were investigated in hydrolysis of AB (100 mM AB in 10 mL of H_2_O) at 25.0 °C. The recyclability and the reusability tests were performed using 120.0 mg and 160.0 mg of Ru/WO_3_ (1.0% wt.) catalysts, respectively. For the former test, 1.0 mmol AB was transferred into the reactor for another run of the hydrolysis when the first run of hydrolysis was completed. For the latter test, the catalyst and the reaction solution were isolated by centrifugation after the first run. The isolated catalyst was redispersed in 10.0 mL of H_2_O in the reaction vessel and a new batch of AB was transferred into this mixture and the second run of the hydrolysis reaction was started again. The same procedure was followed after the second run, third run, and forth run. Note that bare WO_3_ provides no activity in the hydrolysis reaction.

## 3. Results and discussion

**Figure 1:**
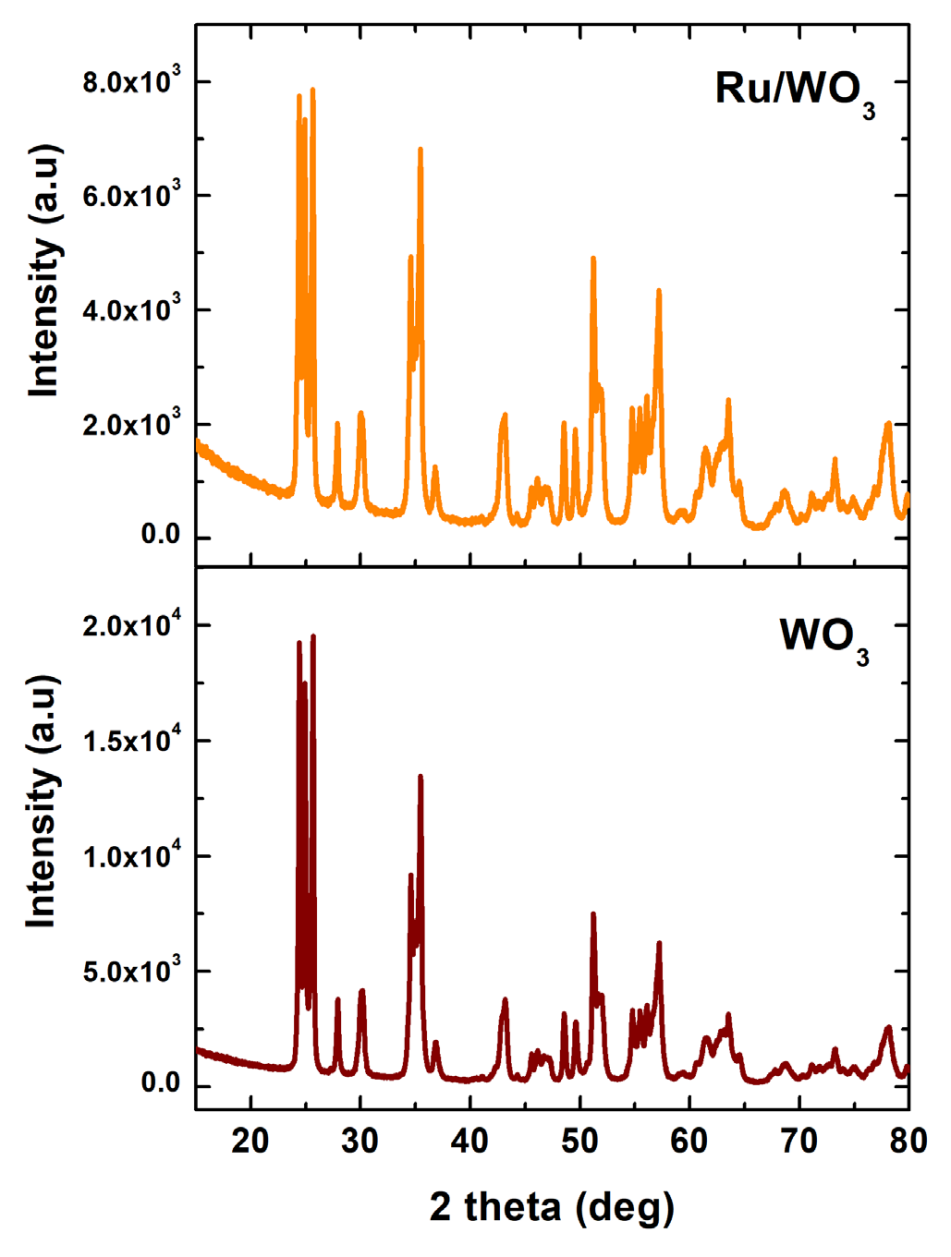
XRD pattern of WO_3_ support and Ru^0^/WO_3_ (1.0% wt. Ru) NPs.

Ruthenium(0) NPs supported on tungsten(VI) oxide powder were successfully prepared from the reduction of Ru^3+^ ions on the surface of support by sodium borohydride. The ruthenium contents of the obtained three Ru^0^/WO_3_ samples were determined by ICP and found to be 1.0%, 2.03%, and 3.01% wt. Ru which were very close to the theoretical values and similar to the values obtained by the EDX surface analysis (vide infra). Consequently, the three samples are denoted as Ru^0^/WO_3_ (1.0% wt. Ru), Ru^0^/WO_3_ (2.0% wt. Ru), and Ru^0^/WO_3_ (3.0% wt. Ru). The obtained Ru^0^/WO_3_ catalysts were characterized by TEM, XRD, FE-SEM, XPS, and EDX techniques. A comparison of XRD patterns of the bare WO_3_ and Ru^0^/WO_3_ samples (Figure 1) indicates that the tungsten(VI) oxide support retains its crystallinity and integrity after the impregnation and reduction of ruthenium(III) ions on the surface; that is, after the ruthenium loading. Note that no diffraction peak is observed for the ruthenium(0) NPs because of the low loading (<3.0% wt. Ru).

**Figure 2:**
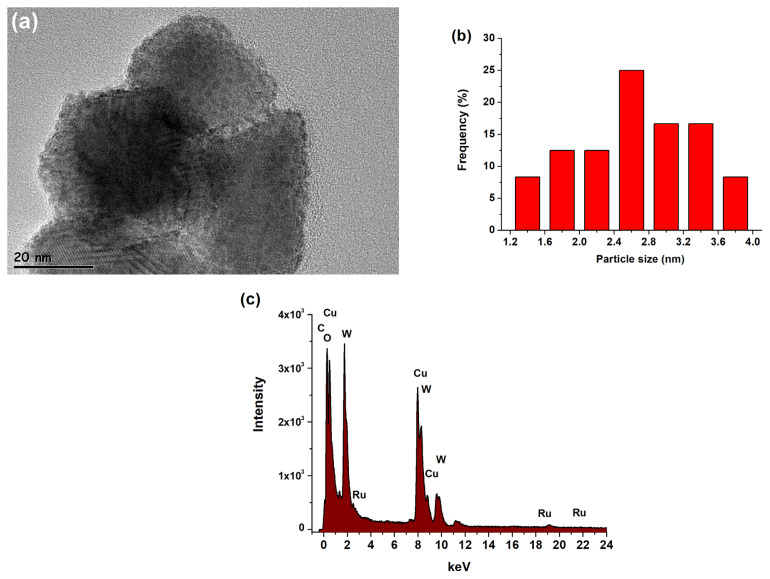
(a) TEM image, (b) histogram for the size distribution, and (c) TEM-EDX spectrum for Ru/WO_3_ (1.0% wt. Ru).

**Figure 3:**
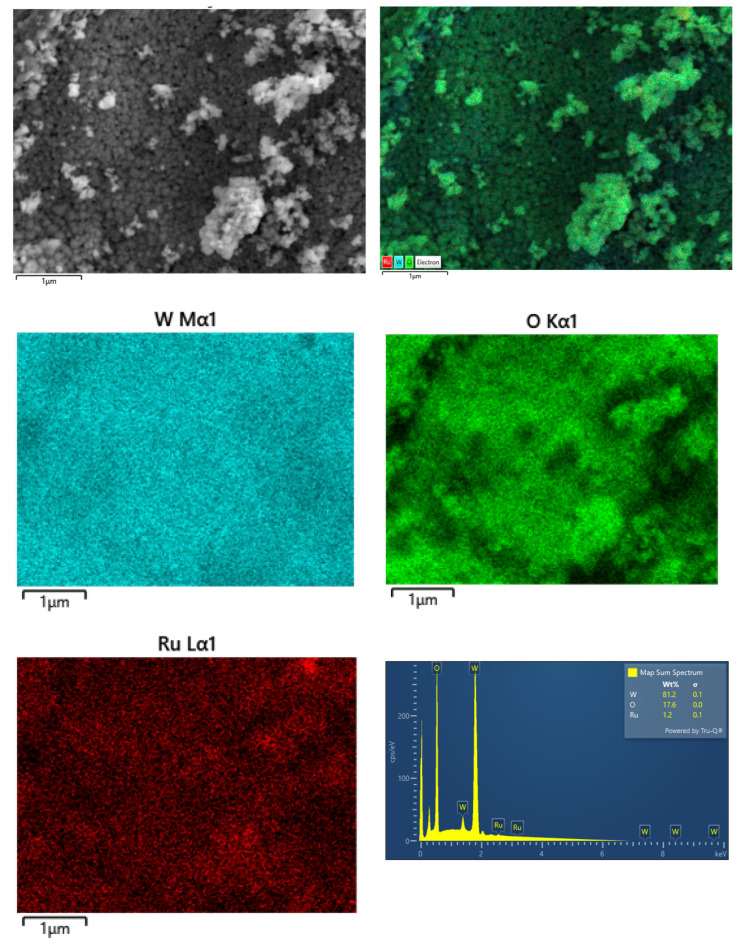
FE-SEM mapping and EDS of Ru/WO_3_ (1.0% wt. Ru) sample. Note that cyan, green, and red colors on the images obtained by electron mapping show W, O, and Ru elements, respectively.

**Figure 4:**
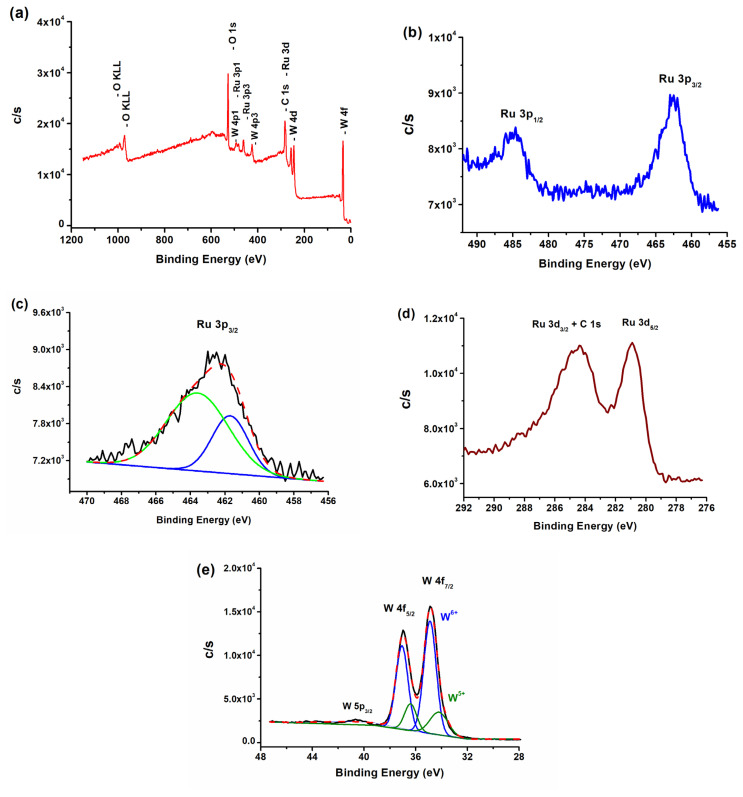
XPS spectra of the Ru^0^/WO_3_ (1.0% wt. Ru) nanocatalyst: (a) Survey scan, (b) high-resolution Ru 3p, (c) high-resolution Ru 3p_3/2_, (d) high-resolution Ru 3d, and (e) high-resolution W 4f.

TEM-EDX (Figure 2), FE-SEM electron mapping (Figure 3), and survey scan XPS (Figure 4) analyses confirm the presence of Ru^0^ NPs well dispersed on the surface of tungsten(VI) oxide support. Furthermore, they show that the sole elements existent in the sample are ruthenium, tungsten, and oxygen. The TEM image of Ru^0^/WO_3_ nanocatalyst with 1.0% wt. Ru loading (Figure 2a) shows the presence of highly dispersed ruthenium(0) NPs on the WO_3_ support. The histogram in Figure 2b shows the particle size distribution in the range 1.5–4.0 nm with a mean diameter of 2.6 nm. XPS was used to investigate the elemental composition of Ru^0^/WO_3_ (1.0% wt. Ru) nanocatalyst as well as the oxidation state of Ru NPs. A survey scan XPS analysis confirms the existence of only O, W, and Ru elements (Figure 4a). High-resolution scans of Ru 3p and Ru 3d XPS are displayed in Figures 4b and 4d, respectively. The Ru 3d_3/2_ peak in Figure 4d unfortunately overlaps with C 1s peak which makes the analysis difficult. High resolution scan Ru 3p XPS in Figure 4b exhibits two intense peaks at 462.6 and 485 eV which are ascribed to the ruthenium(0) 3p_3/2_ and 3p_1/2_ bands, respectively [100,101].

Since the Ru^0^ 3p_1/2_ and W 4p_1/2_ peaks overlap with each other, we could analyze only the Ru^0^ 3p_3/2_ peak in Figure 4c which shows its deconvolution to two well-resolved peaks at 461.7 and 463.5 eV. The higher energy peak at 463.5 eV is assigned to a ruthenium(0) species with slightly higher partial positive charge than the other ruthenium(0) species giving rise to the lower energy peak at 461.7 eV [102]. The observation of two Ru^0^ 3p_3/2_ peaks in Figure 4c implies the presence of two different ruthenium atoms in ruthenium(0) NPs. These two ruthenium(0) species differ from each other by the extend of interaction with the reduced tungsten oxide support. Indeed, the high resolution scan W 4f XPS in Figure 4e gives an undeniable evidence for the reduction of tungsten oxide support as it shows two prominent intense peaks for tungsten(VI) which are readily ascribed to the W^6+^ 4f_7/2_ and 4f_5/2_ bands [103] and more importantly, two weak intensity peaks (recognized by deconvolution) belong to the W^5+^ 4f_7/2_ and 4f_5/2_ bands [104]. Note that the peaks at 34.9 and 37.1 eV are ascribed to W^6+^ while the other two weak intensity peaks at 34.2 and 36.4 eV are assigned to the W^5+^ in Figure 4e. The observation of two peaks for the tungsten(V) species explains the color change observed for the oxide support under the reducing conditions (vide infra).

The WO_3_ support changes its color from light green to blue under the reducing conditions, that is, in the presence of reducing agent (sodium borohydride or ammonia borane). Observation of color change indicates the fractional reduction of tungsten(VI) to tungsten(V) which is demonstrated by the high resolution W 4f XPS in Figure 4e as reported previously [99]. The fractional reduction causes a negative charge building up on the support surface which can affect catalytic activity of ruthenium(0) NPs. Furthermore, the high resolution Ru 3p_3/2_ XPS in Figure 4c exhibits the presence of two kinds of putative ruthenium(0): The one at higher binding energy of 463.5 eV has slightly higher partial positive charge than the other at 461.7 eV. Nearly spherical NPs on the tungsten oxide surface have essentially two kinds of ruthenium atoms; the first kind of ruthenium atoms is in contact with the oxide surface, the second kind is free and does not interact directly with the surface. The reduced tungsten oxide may cause negative charge on the support surface which interacts with the ruthenium atoms in contact with the oxide surface. Charge transfer occurs from ruthenium metal to oxide surface in this metal-support interaction. Thus, the ruthenium atoms of the first kind, which are in direct contact with the support, gain higher partial positive charge through the interaction with the oxide surface than the second kind as shown by the XPS. Hence, the strong interaction between the reducible WO_3_ and ruthenium(0) NPs has an influence on both the stability and activity of Ru^0^/WO_3_ nanocatalyst in releasing 3 equivalent H_2_ per mole of AB in hydrolysis.

**Figure 5:**
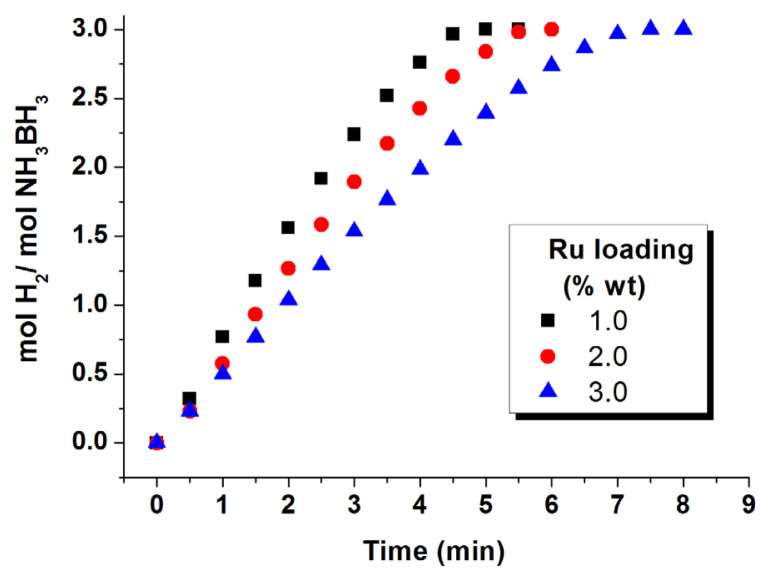
Hydrogen evolution plots (equivalent H_2_ per mole of AB versus time) for the hydrolytic dehydrogenation of ammonia borane starting with Ru^0^/WO_3_ NPs at different ruthenium loadings at 25.0 ± 1.0 °C. [Ru] = 0.6 mM and [AB] = 100 mM.

Catalytic activity of Ru^0^/WO_3_ NPs with different ruthenium contents was tested in H_2_ generation from the hydrolysis of AB. Figure 5 shows the hydrogen generation plots for the hydrolysis of AB (100 mM) at 25.0 °C performed starting with Ru^0^/WO_3_ NPs of three different ruthenium loadings (1.0%, 2.0%, and 3.0% wt. Ru). The H_2_ generation starts immediately and lasts almost linearly until the end of hydrolysis releasing 3 equivalent H_2_ per mole of AB. The immediate start of hydrogen evolution indicates that the catalyst is preformed by borohydride reduction of precursor. TOF values can be computed from the linear portion of hydrogen evolution plots using the formula given in the experimental section (SI). The TOF values of Ru^0^/WO_3_ nanocatalysts with ruthenium contents of 1.0%, 2.0%, and 3.0% are 122, 106, and 83 min^−1^, respectively, for the release of 3 equivalent H_2_ per mole of AB in hydrolysis at 25.0 °C. As expected, the catalytic activity decreases with the increasing metal content [105]. Since the Ru^0^/WO_3_ (1.0% wt. Ru) nanocatalyst shows the maximum activity, it was used for all the other hydrolysis experiments in the present study. The TOF value of Ru^0^/WO_3_ (1.0% wt. Ru) nanocatalyst is found to be comparable to the ones of the ruthenium(0) NPs on the surface of other oxide supports listed in Table.

**Figure 6:**
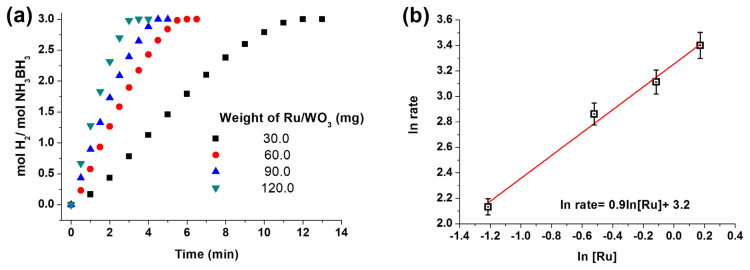
(a) Hydrogen generation plots (mol H_2_/mol H_3_N.BH_3_ versus time) for Ru^0^/WO_3_ NPs depending on catalyst weight in hydrolysis of AB (100 mM) at 25.0 ± 0.1 °C. (b) Hydrogen generation versus ruthenium concentration plot, both axes in logarithmic scale.

Figure 6 shows hydrogen evolution plots recorded during AB hydrolysis starting with Ru^0^/WO_3_ (1.0% wt. Ru) nanocatalyst in various catalyst weight at 25.0 °C. Hydrogen generation rate was determined from the slope of each plot in the linear portion (Figure 6a) and charted vs. ruthenium concentration, both in logarithmic scale. This gives a straight line with a slope of 0.9 specifying that the hydrolysis reaction of AB catalyzed by Ru/WO_3_ (1.0% wt. Ru) NPs is first order with respect to concentration of the ruthenium catalyst (Figure 6b). Since the hydrogen generation occurs linearly almost until the end in all of the hydrogen generation plots, one can conclude that the hydrolysis reaction is zero order with respect to AB concentration.

**Figure 7:**
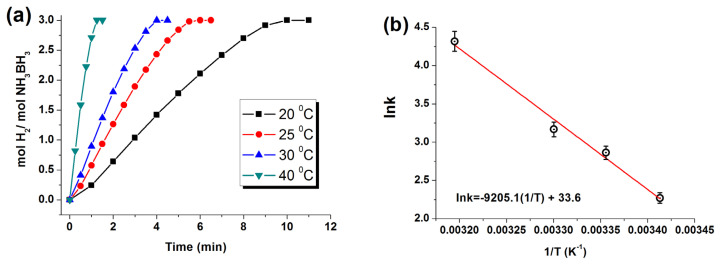
(a) Hydrogen generation plots (mol H_2_ per mole of AB vs. time) during the hydrolysis reaction at various temperatures starting with Ru^0^/WO_3_ (1.0% wt. Ru). [Ru] = 0.60 mM, [AB] = 100 mM. (b) Arrhenius plot for AB hydrolysis catalyzed by Ru^0^/WO_3_ (1.0% wt. Ru).

Hydrolysis reaction of AB was also performed at various temperatures using Ru/WO_3_ (1.0% wt. Ru) nanocatalyst (Figure 7a), where rate constants for the H_2_ evolution were determined from the slope in linear part. The temperature-dependent rate constants could be evaluated by constructing the Arrhenius graph in Figure 7b to calculate the apparent activation energy to be E_a_ = 77 ± 2 kJ mol^−1^ for AB hydrolysis using Ru^0^/WO_3_ (1.0% wt. Ru).

**Figure 8:**
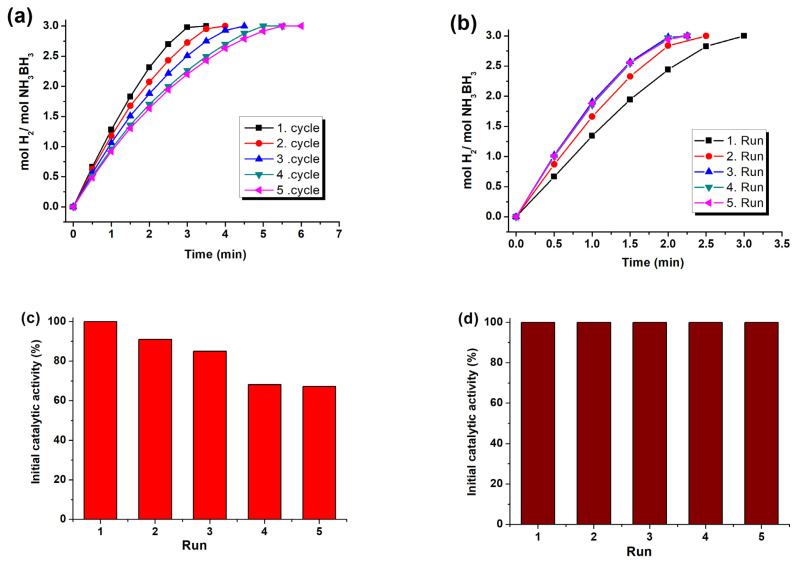
Hydrogen generation plots (mol H_2_ per mole of AB vs. time) in catalytic hydrolysis of AB (100 mM) starting with (a) Ru^0^/WO_3_ (1.18 mM Ru) and (b) Ru^0^/WO_3_ (1.58 mM Ru) from the first to the fifth run at 25.0 °C. Percent initial activity retained in use for (c) the recyclability and (d) the reusability test.

The durability and stability of nanocatalysts can be measured by percentage of the retained initial activity in the consecutive recyclability and reusability tests, respectively. The recyclability check is implemented by addition of a new batch of substrate to the reaction solution after complete hydrolysis in the present run without isolation of catalyst. For testing the reusability of nanocatalysts in AB hydrolysis, the catalyst needs to be isolated from the solution after the completion of reaction and then, placed in a new reactor with new batch of substrate for the following run of hydrolysis. The initial activity retained in each run is recorded and given in percentage [106]. Figure 8 displays the scores of recyclability and reusability tests for the Ru^0^/WO_3_ (1.0% wt. Ru) nanocatalyst. The results of recyclability test in Figures 8a and 8c reveal that the catalyst retains only 67% of initial activity after the 5th cycle. The activity loss during the consecutive cycles is due to deactivation by metaborate ions which are accumulated during the whole reaction. Figures 8b and 8d show the results of reusability tests performed by isolating and then redispersing nanocatalyst in a new AB batch for the next run. There is no activity loss in successive runs. A noteworthy observation is that activity increases on passing from the first run to the second and then to the third run. Then it remains constant. Furthermore, the isolated solution after each run of reusability exhibits no catalytic activity in the hydrolysis of AB, which demonstrates the absence of leaching of ruthenium from the support to the reaction solution. The increase in catalytic activity on going from the first to the third run is likely due to the continuing formation of Ru^0^ NPs by in situ reduction of ruthenium in higher oxidation state which might be formed by air exposure during the isolation and redispersing procedure.

The activity and reusability of ruthenium catalysts plus activation energies for the hydrolytic dehydrogenation of ammonia borane including the ones reported in the previously uncited papers [107–114] are listed in Table. Ru^0^/WO_3_ (1.0% wt. Ru) nanocatalyst provides a descent activity in releasing H_2_ from AB as compared to the literature values. Another noteworthy observation is that there is no correlation between the activation energy and catalytic activity in contrast to the general belief that the decreasing activation energy would correlate with the increasing catalytic activity.

## 4. Conclusions

Ru^0^/WO_3_ (1.0% wt. Ru) nanocatalyst with a mean diameter of 2.6 nm provides descent activity in releasing H_2_ from the hydrolysis of ammonia borane at room temperature, and more importantly, it appears to be reusable as the catalyst retains 100% of initial activity in the fifth run of hydrolysis. Since no leaching is observed for the ruthenium(0) NPs from the oxide surface to the solution, there exists conceivably a strong metal-support interaction between the ruthenium and tungsten oxide. The XPS analyses yield results supporting such a strong interaction between the ruthenium(0) NPs and the reducible tungsten oxide surface.

The Ru^0^/WO_3_ (1.0% wt. Ru) nanocatalyst exhibits unusually high stability. Usually, such a high reusability can be achieved by the magnetically isolable NPs [25], as the nanocatalyst can readily be recovered using an exterior magnet. Although the nonmagnetic Ru^0^/WO_3_ nanocatalysts need to be isolated by filtration or centrifugation, no activity loss is observed even after the 5th run of hydrolysis. Hence, this high reusability of Ru^0^/WO_3_ nanocatalysts can make a noticeable contribution to the utilization efficiency of precious ruthenium in catalyzing the hydrolytic dehydrogenation of ammonia borane.

## Supplementary Material

### Materials

Ammonia borane (NH_3_BH_3_, 97%), ruthenium(III) chloride hydrate (RuCl_3_·3H_2_O, 99.0%), tungsten(VI) oxide (WO_3_, 98%, average particle size of 100 nm, surface area of 9.7 m^2^/g) and sodium borohydride (NaBH_4_, 98%) were purchased from Sigma-Aldrich. Deionized water was distilled by water purification system (Milli-Q System). All glassware and Teflon-coated magnetic stir bars were cleaned with acetone, followed by copious rinsing with distilled water before drying in an oven at 150 °C.

### Instrumentation

Ruthenium content of Ru/WO_3_ samples was determined by the Inductively Coupled Plasma Optical Emission Spectroscopy (ICP-OES, Leeman-Direct Reading Echelle) after each sample was completely dissolved in the mixture of HNO_3_/HCl (1/3 ratio). Transmission electron microscopy (TEM) was performed on a JEM-2100F (JEOL) microscope operating at 200 kV. Samples were examined at magnification between 400 K and 700 K. The X-ray photoelectron spectroscopy (XPS) analysis was performed on a Physical Electronics 5800 spectrometer equipped with a hemispherical analyzer and using monochromatic Al Kα radiation of 1486.6 eV, the X-ray tube working at 15 kV, 350 W and pass energy of 23.5 keV. The binding energy scale was referenced to the C1s signal of 284.5 eV. Powder X-ray diffraction (XRD) patterns were acquired on a Rigaku Mini Flex X-ray diffractometer (radiation source Cu Kα, λ = 0.15418 nm, and scanning rate = 2 min^−^^1^). Field emission scanning electron microscopy (FE-SEM) and energy dispersive spectroscopy (EDS) were performed by using a ZEISS GeminiSEM 500.

### Catalytic activity of Ru/WO_3_ in hydrolysis of AB

The hydrolysis reaction was performed in a jacketed reaction flask (20 mL) containing the Ru^0^/WO_3_ catalyst. The temperature of the reaction was adjusted by circulating water through the jacket of the flask from a constant temperature bath. A graduated glass tube filled with water was connected to the flask to measure the volume of the H_2_ gas generated during the hydrolysis reaction. Next, 1.0 mmol of AB, NH_3_BH_3_, (31.8 mg) was added into the flask and the reaction medium was stirred at 1000 rpm. The volume of H_2_ gas was measured by recording the displacement of water level every 1.0 min at constant atmospheric pressure.

### Turnover frequency (TOF) calculation

Turnover frequency was calculated as described elsewhere [100] by the following equation:


$$\text{TOF}=\frac{n({\text{H}}_{2})}{n(\text{Ru})\times \text{t}}$$

where TOF is the turnover frequency in min^−1^, n(H_2_) is the mole number of collected H_2_ in mmol, n(Ru) is the mole number of total ruthenium in Ru^0^/WO_3_ in mmol, t is the time elapsed for the 40% conversion of ammonia borane. Note that TOF value was not corrected for the fraction of active sites.

## Figures and Tables

**Table:** Apparent activation energy (E_a_) and TOF values for ruthenium-based catalysts reported for AB hydrolysis at 25 °C. ^*^where catalyst was isolated from reaction medium after each of successive run for the next use.

Catalyst	TOF (min^−1^)	E_a_ (kJ mol^−1^)	Reusability % activity retained	Ref.
Ru NPC	813	25	^*^67.3% in 5 run	107
Ru/graphene	600	13	80% in 5 cycle	48
Ru/graphene	100	12	72% in 4 cycle	49
Ru/carbon black	430	35	^*^43.1% in 5 run	47
Ru/AC	235	68	100% in 5 cycle	45
Ru/MWCNT	329	33	^*^41% in 4 run	43
Ru/g-C_3_N_4_	313	37	^*^50% in 4 run	62
Ru/nanodiamond	229	51	^*^40% in 5 run	60
Commercial Ru/C	113	76	-	108
Nanoporous Ru	27	67	67% in 5 cycle	109
Ru/γ-Al_2_O_3_	88	29	-	73
Ru/SiO_2_	200	38	-	65
Ru/SiO_2_-CoFe_2_O_4_	173	46	^*^94% in 10 run	66
Ru/SiO_2_-Fe_3_O_4_	127	54	^*^100% in 5 run	110
Ru/TiO_2_ nanotube	303	46	25% in 4 cycle	74
Ru/TiO_2_	241	70	100% in 3 cycle	76
Ru/TiO_2_ (anatase)	200	87	65% in 5 cycle	75
Ru/TiO_2_ (anatase+rutile)	604	38	-	111
Ru/ZrO_2_	173	58	^*^67% in 5 run	77
Ru/HfO_2_	170	65	^*^75% in 5 run	78
Ru/CeO_2_	361	51	^*^60% in 5 run	79
Ru@S1B-10C	202	24	70% in 5 cycle	83
Ru/SBA-15	316	35	100% in 5 cycle	84
Ru-MIL 53 (Al)	267	34	75% in 4 cycle	86
Ru-MIL 53 (Cr)	261	29	71% in 4 cycle	86
Ru/MCM-41	288	42	75% in 5 cycle	90
Ru-MIL 96	231	48	65% in 5 cycle	88
Ru-MIL-101	178	51	-	112
Ru/HAP	137	58	^*^92% in 5 run	113
Ru/X-NW	135	77	-	80
Ru@ZK-4	90	28	^*^85% in 5 run	114
Ru^0^/WO_3_	122	27	^*^100% in 5 run	This study
